# India's 'gold mine' of ancestral bacilli and the looming TB-HIV pandemic

**DOI:** 10.1186/1476-0711-5-31

**Published:** 2006-12-22

**Authors:** Niyaz Ahmed, Hakan Leblebicioglu

**Affiliations:** 1 Pathogen Evolution Group, Centre for DNA Fingerprinting and Diagnostics (CDFD) Hyderabad, India; 2 Department of Infectious Diseases and Clinical Microbiology, Ondokuz Mayis University Medical School, Samsun, Turkey

## Editorial

It's almost a decade since the whole genome sequence of the tubercle bacillus *M. tuberculosis *was completed. The genome sequence of tuberculosis (TB) bacteria has opened various new avenues for studying biology of TB as well as heralded major funding possibilities for researchers worldwide. However, it is pathetic to realize that we do not have ready a 'promising' new drug target, a 'perfect' vaccine candidate or a 'gold standard' diagnostic marker as yet, for this dreaded pestilence. Countries like India are worst sufferers of such a disappointingly delayed fruit of post-genomic TB research and technology. Nonetheless, significant benefits arising in terms of exploratory genomics have helped the clinical cause of tracking and analyzing the strains of epidemic potential. Gutierrez and colleagues [1] in a recently reported multicentric research based on genotyping of TB strains prevalent in modern day India pointed to reservoirs of the ancestral strains, which continue to predominate throughout the population. Sujatha Narayanan from the Tuberculosis Research Centre in Chennai echoed similar scenario for Tamil Nadu area in the recently held 8^th ^Congress of Molecular Epidemiology and Evolutionary Genetics of Infectious Diseases (MEEGID) at Bangkok in Thailand [2].

*M. tuberculosis *is a millennia old pestilence (perhaps since more than 10, 000 BC) that continues to haunt the population here. Genotyping of patient isolates through the repertoire of variable-number tandem repeats (VNTRs) portrayed *M. tuberculosis *strain diversity in India. A genomic signature, called as tuberculosis specific deletion 1 (TbD1), a chunk of DNA whose presence determines the ancestral *M. tuberculosis *[3], a geno-family of possibly low disseminating strains as compared to some of the very highly spreading and expanding strains such as the Beijing types [4,5]. Predominance of the ancestral strains possibly suggests that India has been the ancient reservoir for TB in the continent [1].

The ancestral strains bear seemingly important benefits for the TB control programs in India. More importantly, as a result of their adaptive evolution, the pathology triggered by them may not be lethal. This analogy derives support from a series of laboratory animal experiments pioneered by D. A. Mitchison in the early seventies showing majority of the South Indian strains caused low grade pathology [6,7]. Given the reportedly highest proportion of ancestral strains in the South, we may hypothesize that Mitchison's 'less virulent' bacteria were most probably the bacilli of ancestral (TbD1+) group. This needs to be further investigated. Also, it is speculated that such strains disseminate less rapidly than the modern types possibly due to a 'genomic load' of unshed TbD1 – thus an advantage for local transmission dynamics. Consequently, these strains might be comparatively less prone to acquisition of resistance to antimicrobials – another advantage.

But the country is now seeing slow but gradual rise of Beijing geno-family of strains that might out-compete the ancestral types. The outcome could be hastened as India is witnessing a steep rise in the number of human immunodeficiency virus (HIV) cases, exceeding South Africa in prevalence, with an estimated 5.7 million cases [8]. Synergy of TB, lead predominantly by the Beijing strains, with HIV, threatens a series of outbreaks in several years to come. With fast spreading HIV, local advantages due to ancestral bacilli, in terms of adaptation, and possibly 'reduced virulence' might be ruined; HIV through depleting the host immune cells disregards any such advantages.

Although Beijing strains are not an immediate threat, there is a danger that they might predominate in due course if their dissemination dynamics change with enhanced HIV transmission. Also, their link with several outbreaks of drug resistant tuberculosis worldwide suggests a genetically encoded propensity to acquire multi-drug resistance as they spread and cross through their host. It is clear that there is an ongoing transmission of Beijing in India for which published reference is currently meager, but individual labs have records based on genotyping on limited to moderately large sample sizes. Nonetheless, there are incidences of Beijing being reported up to as high as 30% from Mumbai [9], but it is still 8–10% in Delhi [1,10]. Transmission of Beijing strains is more likely to be facilitated with recent economic activity due to a boom in the Information Technology (IT) and communication sectors where affordable air-travel has facilitated frequent movement of especially younger population, across cities.

It has been widely believed that India with its vast human resource in healthcare, with DOTS coverage penetrating almost countrywide, and a large national TB control program, is all set to tackle the pestilence. We caution, to prepare for the threat of institutionalized outbreaks perpetuated by newly emerging and expanding strains in synergy with HIV, that is probably looming large. It is true that dedicated strain-typing facilities to monitor emergence and preponderance of aggressive and non-aggressive strains are almost non-existent here and epidemic-forecasting systems therefore, would be difficult and tough to devise. In view of this, it will be highly appropriate to monitor carefully the comparative spread dynamics of *M. tuberculosis *lineages in different settings through a nationwide surveillance and response strategy on the lines of the Centers for Disease Control and Prevention (CDC), of the USA.

A European equivalent of CDC has been recently shaped in Stockholm, thanks to the efforts championed by Michel Tibayrenc, from the IRD in Montpellier, France. It took quite long for Tibayrenc to muster the support of several scientific organizations, attention of the policy makers, and finally of politicians before his idea of European CDC was approved in April 2004 under the new European legislation. After European CDC, do we need an Indian CDC? The answer is definitely 'yes' because India simply does not have a sophisticated and focussed public health laboratory system for dynamic epidemic surveillance and disease forecasting. In a country that requires more effective surveillance of disease, better responses to epidemics, and better disease prevention, such a new Centre, a possible networking hub of hundreds of public health laboratories, will play a central role in the context of preventing the HIV-TB pandemic.

## References

[B1] Gutierrez MC, Ahmed N, Willery E, Narayanan S, Hasnain SE, Chauhan DS, Katoch VM, Vincent V, Locht C, Supply P (2006). Predominance of ancestral lineages of Mycobacterium tuberculosis in India. Emerg Infect Dis.

[B2] Narayanan S, Deriemer K, Gagneux S, Hari L, Tsolaki A, Rajasekhar S, Narayanan PR, Small PM (2006). Mycobacterium tuberculosis isolates from South India belongs to an ancient lineage. Proceedings of the 8th International Meeting on Molecular Epidemiology and Evolutionary Genetics in Infectious Diseases, Bangkok, Thailand, November 30-December 2.

[B3] Brosch R, Gordon SV, Marmiesse M, Brodin P, Buchrieser C, Eiglmeier K, Garnier T, Gutierrez C, Hewinson G, Kremer K, Parsons LM, Pym AS, Samper S, van Soolingen D, Cole ST (2002). A new evolutionary scenario for the Mycobacterium tuberculosis complex. Proc Natl Acad Sci USA.

[B4] Rad ME, Bifani P, Martin C, Kremer K, Samper S, Rauzier J, Kreiswirth B, Blazquez J, Jouan M, van Soolingen D, Gicquel B (2003). Mutations in putative mutator genes of Mycobacterium tuberculosis strains of the W-Beijing family. Emerg Infect Dis.

[B5] Glynn JR, Whiteley J, Bifani PJ, Kremer K, van Soolingen D (2002). Worldwide occurrence of Beijing/W strains of Mycobacterium tuberculosis: a systematic review. Emerg Infect Dis.

[B6] Bhatia AL, Csillag A, Mitchison DA, Selkon JB, Somasundaram PR, Subbaiah TV (1961). The virulence in the guinea-pig of tubercle bacilli isolated before treatment from South Indian patients with pulmonary tuberculosis. 2. Comparison with virulence of tubercle bacilli from British patients. Bull World Health Organ.

[B7] Mitchison DA (1964). The virulence of tubercle bacilli from patients with pulmonary tuberculosis in India and other countries. Bull Int Union Tuberc.

[B8] (2006). People living with HIV/AIDS (adults and children) global data. http://www.globalhealthfacts.com.

[B9] Almeida D, Rodrigues C, Ashavaid TF, Lalvani A, Udwaida ZF, Mehta A (2005). High incidence of the Beijing genotype among multidrug-resistant isolates of Mycobacterium tuberculosis in a tertiary care center in Mumbai, India. Clin Infect Dis.

[B10] Singh UB, Suresh N, Bhanu NV, Arora J, Pant H, Sinha S, Aggarwal RC, Singh S, Pande JN, Sola C, Rastogi N, Seth P (2004). Predominant tuberculosis spoligotypes, Delhi, India. Emerg Infect Dis.

